# An Analysis of the Ethical, Economic, and Environmental Aspects of Entomophagy

**DOI:** 10.7759/cureus.26863

**Published:** 2022-07-14

**Authors:** Jofre Illa, Oriol Yuguero

**Affiliations:** 1 Faculty of Health Sciences, Universitat de Lleida, Lleida, ESP; 2 Faculty of Medicine, Universitat de Lleida, Lleida, ESP

**Keywords:** sustainability, climate change, nutrition, ethics, entomophagy

## Abstract

Population growth is causing a high demand for meat products, which, coupled with the current climate crisis, has fueled research into alternative protein sources. This review discusses the role of edible insects as an alternative protein source to complement our diet. We compare nutritional, environmental, economic, and food safety aspects between edible insects and current protein sources and conclude with a discussion on ethical considerations. Edible insects are a good protein source, with a higher average protein content than most protein-rich products we consume today. In addition, they provide fiber, fats such as monounsaturated fatty acids (MUFAs) and polyunsaturated fatty acids (PUFAs), and essential amino acids and are also rich in some minerals and vitamins. Product safety is yet to be studied; however, they have a much lower environmental impact than other intensive livestock products. Moreover, the production of edible insects is not expected to be expensive. The consumption of edible insects is a good alternative to conventional animal foods in modern times: a major climate crisis accompanied by numerous societal inequalities due to population growth.

## Introduction and background

The growing demand for meat and the limited agricultural land for livestock farming have fueled research into alternative protein sources. An accurate estimate of the number of edible insect species worldwide is still pending. However, the literature published in various world regions has identified more than 1,900 edible insect species. Most of the insects currently consumed are species from the orders Coleoptera (31%), Lepidoptera (18%), Hymenoptera (14%), Orthoptera (13%), and Hemiptera (10%) [1]. According to the International Feed Industry Federation (IFIF), the world population will reach 10,000 million by 2050 [2], doubling the demand for animal protein. As a result, the increase in pork and poultry consumption will reach 105% and 175%, respectively, making edible insects a critical component in the food chain.

The Food and Agriculture Organization (FAO) notes the limited supply of conventional animal foods and advocates the importance of finding alternatives [3]. The European Commission’s European Food Safety Authority (EFSA) authorized the safe consumption of the yellow mealworm in January 2021 and the common cricket in July of the same year. Therefore, the consumption by humans of these insects is risk-free. However, many societies view these products as dietary taboos, thus presenting social and cultural obstacles [4].

Entomophagy consists of eating insects for food. It comes from the Greek words “entomon,” meaning “insect,” and “phagein,” meaning “to eat.” Combining the two, entomophagy means “to eat insects” [5]. Eating insects in many regions and countries during human evolution was common. Insects have historically been and are still important sources of nutrients in some parts of the world. The main hindrance to the human consumption of insects is culture. Since our ideology of animals influences our perception of insects as food more than their nutritional value, cultural beliefs pose a problem regarding including insects in the diet.

Insects should be promoted as a nutrient source rather than a new type of food [6]. Currently, in most Western societies, protein continues to be supplied by animals such as cattle, pigs, and chickens and protein-rich foods such as pulses. Insects are synonymous with annoyance; for example, mosquitoes and flies are commonplace in the house; the former bite us, termites destroy wood, and some insects eat our food, causing a feeling of disgust [5].

This review discusses whether protein from edible insects can be an alternative to today’s protein. Different aspects are assessed and compared between these protein sources, such as their composition, food safety, and the cost and environmental impact of their production. Finally, ethical issues are discussed, considering the world’s current social and environmental situation.

## Review

Nutritional value of insects

Insects are a traditional food source in many parts of the world. They satisfactorily provide energy, proteins, and the required amino acids of living beings, and they have a high content of monounsaturated fatty acids (MUFAs) and polyunsaturated fatty acids (PUFAs). In addition, they are rich in various micronutrients such as copper, iron, magnesium, manganese, phosphorus, selenium, zinc, riboflavin, pantothenic acid, biotin, and, in some cases, folic acid [7].

Proteins

The protein/body weight ratio is very high in all insects, with an average higher than that of beef, pork, or protein-rich vegetables. In addition, many species approach the values of fish, currently the most protein-rich food in our diet [8]. Protein content ranges from 7% to 91%, and many species contain approximately 60% of protein. Protein digestibility varies greatly, as the cuticular protein is bound to chitin, a polysaccharide, and a component of the insect’s exoskeleton.

Edible insects generally meet the WHO requirements for amino acids, with high levels of phenylalanine + tyrosine, and are sometimes rich in tryptophan, lysine, and threonine [9]. Therefore, they are high-quality proteins because they contain essential amino acids, which our bodies cannot synthesize and must receive from the diet [10]. The protein content of edible insects ranges from 400 to 750 g/kg dry weight, while that of eggs and milk is 540.7 g/kg and 302.8 g/kg, respectively [11].

Lipids

After proteins, fat accounts for the second most important part of the nutrient composition of edible insects, ranging from 13% to 33% [12]. The fatty acid content and lipid composition of insects are related to species, sex, stage of life, diet, environmental temperature, and migratory flight [6]. Insect fatty acids are comparable to those of poultry and fish in unsaturation levels but contain more PUFAs [12].

Carbohydrates

Like most animals, edible insects are high in proteins and lipids but low in carbohydrates. However, insects are a special animal group in terms of dietary fiber [13]. Insects can have up to 10% fiber, the most common form being chitin. This nitrogen-based carbohydrate is found in the exoskeleton of most insects as a long-chain polymer of N-acetylglucosamine. The chitin content ranges from 2.7 to 49.8 mg/kg in fresh weight and 11.6 to 137.2 mg/kg in dry matter. It is unclear whether chitin can be considered a positive or a negative food component in insects. Some tests suggest that it is beneficial as it is an antioxidant, has anti-cancer properties, and is anti-inflammatory. Conversely, some say chitin can have antinutritional effects because it can bind different macromolecules that make them indigestible in the intestine [14]. Insect carbohydrates exist primarily in two forms: chitin and glycogen. The former is a major component of the exoskeleton, while the latter is a source of energy stored in muscle cells and tissues. The average carbohydrate content of edible insects ranges from 6.71% to 15.98% [3].

Micronutrients

As for vitamins, insects are generally low in retinol but are high in riboflavin, pantothenic acid, biotin, and, in some cases, folic acid [11]. Their micronutrient content presents high amounts of potassium, calcium, iron, magnesium, and selenium. Specifically, they contain more iron and calcium than beef, pork, and chicken. For example, 100 g of caterpillars provides 335% of the recommended minimum iron intake.

Regarding vitamin B12, different species have been studied, such as the yellow mealworm, grasshopper, cricket, and cockroach. The values for this vitamin in these different insects ranged from 0.84 to 13.21 µg/100 g dry weight. Therefore, they are within the range of other foods of animal origin, such as pork (1.0 µg/100 g), or fish, such as mackerel (9.0 µg/100 g), confirming that insects are an excellent source of vitamin B12 and can contribute significantly to human nutritional needs [15]. Table 1 shows a comparison of the larva of *Tenebrio molitor*, better known as the yellow mealworm, with other high-protein products such as pork, beef, chicken, egg, milk, lentils, or soya [12,16-19]. This type of edible insect has been chosen since the EFSA states that it does not pose any safety problems and contains high protein content, making it one of the insects most used for human consumption [20].

**Table 1:** Summary table of the nutrients found in Tenebrio molitor larva versus other high-protein products SFA: saturated fatty acid, MFA: monounsaturated fatty acid, PFA: polyunsaturated fatty acid

Nutrients	Tenebrio molitor larva	Pork	Beef	Chicken	Egg	Milk	Lentils	Soya
Kcal/100 g	247	155	112	125	150	65.6	327	374
Proteins (g/100 g)	25	18.89	20.1	17.8	12.5	3.3	23	35.9
Fats (g/100 g)	12.9	7.05	3.5	6	11.1	3.6	1.8	18.6
Fiber (g/100 g)	3.52	-	-	-	-	-	11.7	15.7
Cholesterol (g/100 g)	51.3	69	59	84	385	14	-	-
Histidine (mg/g prot)	31.6	32	38.1	41.5	22.1	28.9	15	32.8
Isoleucine (mg/g prot)	50.3	49	39.4	55.1	34.6	62	30	45.7
Leucine (mg/g prot)	106.4	75	92.2	70.45	83.9	103	59	81.3
Lysine (mg/g prot)	54.5	78	95.9	90.34	91.4	87.5	45	82.7
Methionine (mg/g prot)	12.8	25	25	28.23	34.9	30.3	16	33.05
Cysteine (mg/g prot)	8.6	13	13	12.5	24	7.8	6	24.1
Threonine (mg/g prot)	41.8	51	51	40.62	47.8	46.2	23	30.05
Tryptophan (mg/g prot)	8	13	11	16	17	15.6	6	14
Calcium (mg/100 g)	47.18	8	5	14	57	121	56	240
Potassium (mg/100 g)	761.54	370	290	320	130	150	737	1730
Magnesium (mg/100 g)	221.54	22	22	23	12	12	78	250
Phosphorous (mg/100 g)	697.44	170	210	130	200	92	240	660
Sodium (mg/100 g)	125.38	76	88	81	140	50	12	5
Iron (mg/100 g)	5.51	1.5	5	1	1.9	0.1	7.1	9.7
Vitamin B6 (mg)	1.21	0.45	0.23	0.42	0.12	0.04	0.6	0.38
Vitamin B9 (µg)	137	5	4	12	50	5	35	370
Vitamin B12 (µg)	0.3	3	13	0.3	2.5	0.3	-	-
Vitamin B2 (mg)	1.21	0.2	0.8	0.15	0.47	0.18	0.2	0.27
Vitamin B3 (mg)	4.1	8.7	6.3	14	3.8	0.8	5.6	7.7
SFA (g)	2.32	2.7	1.7	0.76	3.1	2	0.33	2.3
MFA (g)	2.51	3.5	0.9	1.3	4	0.93	-	1.5
PFA (g)	5.85	1.3	0.1	0.52	1.7	0.09	1	9.1
Cis (g)	0.33	4.6	-	1.8	5.5	0.85	-	-
Trans (g)	0.26	0.17	-	0.02	0.04	0.15	-	-

Food safety

Food safety is one of the most critical factors. Food safety is of special importance when dealing with new food sources. In the context of edible insects, there are four ways through which food safety risks can arise: the insect itself could be toxic, the insect could have acquired toxic substances or pathogens during its life cycle, the insect could become spoiled after harvest, and consumers could experience an allergic reaction to the insect [9].

Mistakes made in the livestock industry should serve as a lesson for controlling insect diseases, such as the use of antibiotics. Disease management strategies should be preventive. The processed product must ensure the safety of the product and preserve its nutritional value. Conservation methods, such as UV rays, pH, or high pressure, should be developed to eliminate possible contaminants [21]. The insect microbiota is highly complex. Apart from the surface of the body and the parts of the mouth, the main habitat of microorganisms is the intestine.

Using insects as food leads to potential microbiological risks because insects can be vectors for pathogenic microorganisms for humans, animals, and plants [22]. They may offer a compatible environment for the growth or survival of bacteria if inadequate treatment measures are employed [23]. The pathogens that can be transmitted are viruses, bacteria, protozoa, fungi, and other parasites. However, insect-specific pathogenic microorganisms are considered harmless to humans due to their high degree of tissue tropism, so they can probably only colonize insect cells or tissues. Still, there are exceptions in some representatives of the genus *Rickettsia*.

More studies are needed to assess and analyze this aspect. Therefore, the transmission of prions to animals and humans by consuming insects contaminated with food containing prions cannot be ruled out and could be considered when deciding the type of fodder used for insect breeding [24]. Insects can act as vectors. Proper heat treatment before consumption can eliminate most microbiological hazards and is effective, particularly against Enterobacteriaceae, while bacterial spores are unaffected.

Particular attention should be paid to storing processed products and domestic processing of fresh insects [25]. Proper hygiene must be applied using handling and storage techniques, such as correct temperature and packaging [25]. Proper management of insects with potential spore content requires storage at a temperature of 5°C-7°C. This temperature is also suitable for preventing the deterioration of boiled insects, which remain stable for more than two weeks [25].

Environmental impact

Edible insects are among the environmentally friendly sources of proteins [21]. Edible insects are being promoted as an alternative source of protein; however, the major challenge will be creating sustainable production systems that will safeguard the environment and ensure food safety and security. Among all insects, only cockroaches, termites, and beetles produce CH4, which originates from the bacterial fermentation of Methanobacteriaceae in the gut [26]. The difference in the environmental impact of pig, poultry, and beef products is due to three main factors: enteric CH4 production, reproduction rate, and food conversion efficiency. The yellow mealworm does not produce CH4. In addition, it has a high reproduction rate since the female *T. molitor* produces 160 eggs in her lifetime. In addition, the maturation period is short, as *T. molitor* reaches adulthood in 10 weeks.

Food conversion efficiency depends, among other things, on the diet supplied. The food conversion ratio (FCR) of yellow mealworm concentrates (kg/kg fresh weight) is similar to the values reported for chickens but lower than for pigs and cattle [27]. Studies have compared different variables between insects and animal meat, and it has been observed that, in general, edible insects have a far lower environmental impact than livestock farming. Caterpillar, locust, and cricket larvae emit 100 times fewer emissions and 10 times less ammonia than cattle and pigs. If insects were bred and consumed instead of cows, the current greenhouse gas emissions would be reduced by 10% [28].

The global warming potential (GWP) index of yellow mealworms per kg of edible protein is low compared to other products such as milk (1.51-3.87 higher), chicken (1.32-2.67 higher), pork (1.51-3.87 higher), or beef (5.52-12.51 higher). Energy use in the production of the yellow mealworm per kg of edible protein is higher than that of milk (20%-83% of the value for the yellow mealworm) or chicken (46%-88%), similar to pork (55%-137% of the value for the yellow mealworm) and lower than that of beef. The yellow mealworm is poikilothermic and depends on suitable environmental temperatures for its growth and development. When ambient temperatures are low, they require warming, which increases energy consumption. Mitigation measures are being investigated; the largest larvae in this system produce a surplus of metabolic heat, which could be used to warm small larvae that require heat.

The land use of the production system described was very low compared to that of milk (1.81-3.23 times higher), poultry such as chicken (2.30-2.85 times higher), pork (2.57-3.49 times higher), and beef (7.89-14.12 times higher) [27]. The production of insects does not require a large area of land, and water use is minimal. The area of land needed to produce the same amount of protein has been estimated to be approximately 1 ha for yellow mealworms, 2-3.5 ha for pigs or poultry, and 10 ha for cattle.

The replacement of meat with insects as the main source of protein could lead to the abandonment of 2,700 Mha of meadows and 100 Mha of farmland, which would result in large carbon sequestration of vegetation. In addition, nitrous oxide and methane emissions would decrease substantially [29]. The growing demand for water worldwide threatens biodiversity, food production, and other vital human needs. For example, yellow mealworms are more drought-resistant than cattle [26]. Regarding digestible biomass, while insects generally reach 80%, other meat products contain only about half, between 40% and 55%. Therefore, in conclusion, edible insects have a lower environmental impact than other animals such as cattle or pigs.

Economic aspects

In developing countries, edible insects are often sold as street food [30]. The economic impact of these markets is underestimated or neglected. The market for edible insects in Western countries is driven by demand from immigrant communities from Africa and Asia and by the development of the exotic food market. It is not easy to predict the future of the edible insect market. Some studies say that the edible insect market will exceed $710 million by 2024. Analysis by different regions of the world revealed that the demand in the North American market could record more than 43.5% growth by 2024. The market for edible insects is favored by the growing demand for high-protein diets and an aversion to processed foods. Furthermore, the Asia-Pacific market could exceed $270 million by 2024. In addition, edible insects are used as food supplements in the manufacture of desserts, smoothies, cookies, and bread due to their high protein content. Moreover, the European market is led by Germany and France and should grow by more than 43% by 2024. A 2016 report estimated that 312 of the 943 million tonnes of proteins consumed in 2054 would be accounted for by alternative proteins other than meat and seafood. Of these, 37 million tonnes will be of insect origin [31].

Moreover, indigenous knowledge systems can be used to build resilience to climate change and increase the production of edible insects. In addition, the knowledge of these groups in the production of insects for consumption can be a stimulus for the economy of certain countries [32]. In addition, it has been seen that the consumption of insects in some European countries, such as the Netherlands, can pose an economic challenge and a new concept for sustainable food industry [33].

Based on a review of the literature done by Żuk-Gołaszewska et al. [34], they concluded that edible insect farming can be a viable business sector that significantly contributes to the overall sustainability of food systems if appropriate regulations are introduced and food safety standards are guaranteed. However, the success of the edible insect industry also requires consumer acceptance of entomophagy, which is rather low in Western societies.

Ethical aspects

Community Nutrition

The consumption of insects as food is affected by factors such as culture and religion. These results might explain the geographical distribution of insect consumption. In addition, there are two different psychological reactions to insects as food. In countries where entomophagy is practiced, insects are not considered a valuable source of nutrients, while in Western cultures, insects are considered dirty, repulsive, and hazardous. In Western societies, only 12.8% of men and 6.3% of women reported being likely to use insects as a substitute for meat [35]. In fact, an Italian study suggests that the introduction of contextual cultural information about insects as a food source may help preclude a priori false assumptions regarding entomophagy [36].

Rapid population growth, environmental pollution, and poor food distribution over the past few years have led to nutrition experts and large sections of the population questioning whether there will be enough food for everyone. Over the past 20 years, food production has lagged behind population growth in many countries, especially in Africa, where a reduction in food production per person has been witnessed in 31 of the 45 countries on the continent. Moreover, the scarce water supply limits development in general and food production in particular [37].

It does not look like the future risks facing agriculture will decrease. On the contrary, we currently perceive that they are set to increase, among other reasons, due to climate change. Global warming leads to the widespread opinion that, based on current knowledge, the future climate will be different from today’s, and no activity is more climate-dependent than agriculture, which must adapt to the new climate [38].

The world’s population has trebled in the last century, and the amount of water used has increased sixfold. Approximately 3,800 million people, or almost two-thirds of the world’s population, live in countries with low incomes and food shortages. In these countries, millions of people experience hunger and malnutrition. Food production is influenced by several factors, among which are limited agricultural land since that which is available is in use, the size of farms is contracting, leading to the land being distributed in small, underproductive parcels, and the soil is being degraded due to irrigation problems.

Worldwide, some 825 million people are chronically malnourished, according to a recent estimate by the FAO, and most of these live in countries with low income and food shortages and, at the same time, the highest population growth rates. By 2050, some 6,000 million people will be living in countries currently experiencing food shortages. Winning what experts call “the food race” requires a coordinated system to increase agricultural production, improve food distribution and resource stewardship, and control population growth by providing family planning services, education, and healthcare, all of which are essential to improving people’s welfare and thus promoting productivity and the sustainable use of resources.

A second “green revolution” in agriculture, to stimulate food production and feed the growing population, like in the 1970s, is needed to achieve this goal. An alternative, notwithstanding what has already been mentioned, to help solve this problem are the major technological breakthroughs happening today, which might not only cover the demand for food in the future but also completely change the approach to food in just a few years [39].

Given the predicted scarcity of traditional resources soon, the food of the future is unknown; however, new options are emerging. Insects could constitute a good alternative as food in the future due to their high protein content, which is essential in our diet, their ease of digestion, their ease of conservation, their high reproductive potential, forming large populations in a short time, and their great environmental adaptability, both on land and in water [40].

It is for this reason that, in the face of food shortages and the search for nutritional resources that can be sustainable and accessible, we believe that entomophagy must be seriously considered. In addition, there is a duty to ensure that the consumption of insects is safe and studied as a viable alternative in the short term, especially in developed countries where there is an important debate about the environmental impact of traditional livestock farming. Can we continue promoting food industries that generate methane when we know that there are balanced and sustainable alternatives? It is evident that we cannot go from current consumption to insect consumption, but a gradual change should be promoted.

## Conclusions

The consumption of edible insects may greatly help, given the major climate crisis we are experiencing and the numerous existing societal inequalities. In addition to their favorable nutritional characteristics, insects provide ecological and economic benefits over conventional animal production and, therefore, may constitute an alternative or a supplement. Economic and social inequalities are a reality in today’s world and have increased in recent years. These inequalities are not just between continents but can be found within each continent and even within countries. These nutritional and social threats include the negative impact of climate change. In today’s climate crisis, governments should provide economic assistance and give importance to sustainable production to reverse the current situation and prepare for what the future might hold in store for us. In this case, the edible insect is a product with a low environmental impact that supports a secular and sustainable economy, leaving behind many of the problems we are encountering with animal production, such as greenhouse gases, scarce water resources, and ammonia pollution, and the lack of farmland.
